# Peculiarities of spirometry in pediatrics

**DOI:** 10.36416/1806-3756/e20250165

**Published:** 2025-07-22

**Authors:** Mario Arturo Flores-Valadez, Irlanda Alvarado-Amador, Laura Gochicoa-Rangel

**Affiliations:** 1. Instituto Nacional de Enfermedades Respiratorias - INER - Ismael Cosío Villegas, Ciudad de Mexico, Mexico.

We face several challenges when evaluating lung function in pediatric patients with respiratory symptoms. On the one hand, their chest wall muscle strength is not enough to maintain low lung volumes (at residual volume), and this is further limited by the fact that lung elastic recoil is increased in children,^1^ thus hindering the forced expiratory maneuver of spirometry; on the other hand, communication and interaction with pediatric patients are essential in order to achieve results that meet the current quality standards for spirometry.^2^ ^,^ ^3^

In order to perform spirometry on a child, we must consider that the laboratory environment should not cause any distractions or overstimulate the child; the calmness and patience of the personnel are essential: better results are achieved if the child perceives the test as a game or becomes familiar with the equipment.^1^ ^,^ ^3^

Multiple devices have developed animations that invite patients to perform a forced expiratory maneuver and encourage them to complete it; however, certain technical factors should be taken into consideration when choosing the best equipment for preschool children. The dead space of the equipment should be minimized (< 2 ml/kg of weight) because it can influence the results. In cooperative children, performing some maneuvers at tidal volume before a forced expiratory maneuver can lead to better results.^1^

In younger children, reduced respiratory muscle strength and increased lung elastic recoil limit exhalation time. Therefore, FEV_0.5_ and FEV_0.75_ have been used in order to assess obstruction in children in the 3- to 5-year age bracket who cannot achieve FEV_1_, with varying degrees of success (between 39% and 70% for FEV_0.5_ and between 9% and 44% for FEV_0.75_), supporting the usefulness of the forced expiratory maneuver in preschool children.^1^ ^,^ ^4^

**Figure 1:**
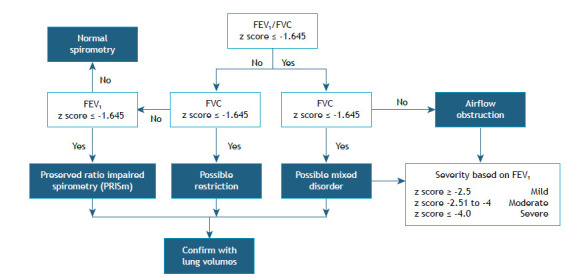
Interpretation of spirometry in pediatric patients.

In children in whom FEV_1_ and FVC are acceptable, the FEV_1_/FVC ratio will determine airflow obstruction on the basis of a z score of < −1.645, based on an equation that is appropriate for the study population. Obstruction should be graded on the basis of the FEV_1_ z score. If an FVC is adequately performed and FVC is decreased, it should ideally be correlated with lung volumes in order to diagnose a restrictive pattern (FVC ≤ −1.645), a mixed pattern (FEV_1_/FVC and FVC ≤ −1.645), or a preserved ratio impaired spirometry pattern (if only FEV_1_ is affected; Figure 1).^5^

Evaluating spirometry after administration of a bronchodilator is particularly important in children, whose results can sometimes be within normal limits or be achieved through a less than perfect technique. A significant change as established in the latest European Respiratory Society/American Thoracic Society technical standard (a 10% change in percent predicted FEV_1_ or FVC) can guide the therapeutic approach to be used in preschool children with respiratory symptoms.^5^

In conclusion, spirometry is feasible even in preschool children. With the appropriate personnel and by adapting simple laboratory conditions and equipment, reliable results can be obtained for the diagnosis and follow-up of children with respiratory symptoms. 
